# The complete chloroplast genome sequence of *Oxalis corniculata* (L.)

**DOI:** 10.1080/23802359.2021.1878950

**Published:** 2021-04-08

**Authors:** Lijing Chen, Bingzheng Jiang, Yue Miao, Ziji Liu, Shangqian Xie, Peng Ling, Jie Zhu

**Affiliations:** a College of Horticulture, Hainan University, Haikou, China; b Ministry of Education, Key Laboratory of Genetics and Germplasm Innovation of Tropical Special Forest Trees and Ornamental Plants (Hainan University), Haikou, China; c Tropical Crops Genetic Resources Institute, Chinese Academy of Tropical Agricultural Sciences, Haikou, China

**Keywords:** *Oxalis corniculata*, chloroplast genome, phylogenetic analysis, Illumina Sequencing

## Abstract

*Oxalis corniculata* L. is a perennial herb with a world-wide distribution. In this study, we sequenced the complete chloroplast genome of *O. corniculata*, which exhibited a circular genome of 155,182 bp in length with 37.5% GC content. The chloroplast genome contained a canonical quadripartite structure with a large single copy (LSC) region of 83,936 bp, a small single copy (SSC) region of 17,048 bp and a pair of 25,581 bp inverted repeats (IRs). A total of 108 unique genes, including 76 protein-coding genes (PCGs), 28 tRNA genes and four rRNA genes were found in this chloroplast genome. The phylogenetic tree was constructed based on *O. corniculata* and other 11 chloroplast genome sequences, which showed that *O. corniculata* was closely grouped with of *O. corymbosa* and *O. drummondii*.

*Oxalis corniculata* L., a perennial herb with unique leaf shape and bright yellow flowers, is one of the widely planted groundcover plants in the garden (Doust et al. 1985). In addition, the whole plant of *O. corniculate* is well-known as medicine, which is used in the treatment of infections, dysentery, diarrhea, skin diseases and insect bites (Raghavendra et al. 2006; Rehman et al. 2015). Here, we report the chloroplast genome of *O. corniculata* in the first time and aim to provide genomic resources for this species.

Total genomic DNA of *O. corniculata* was isolated from the fresh leaves collected in the garden of No.11 dormitory of Hainan University, China (N20°03′19.00″, E110°19′42.08″) following the standard protocol of DNA Extraction Kit (TIANGEN) and stored at −80 °C in the Key Laboratory of Genetics and Germplasm Innovation of Tropical Special Forest Trees and Ornamental Plants of Hainan University. Paired-end (150 bp) sequencing was sequenced by an Illumina HiSeq X-Ten platform. Approximately, 3.68 Gb clean data were obtained and used to assemble the complete chloroplast genomic using GetOrganelle pipeline v1.6.0 (Jin et al. 2018). The complete chloroplast genome of *Oxalis Corymbosa* (GenBank accession No. NC_048890) was used as the reference to annotate the chloroplast genome with the GeSeq (Tillich et al. 2017). The Geneious R11 (Kearse et al. 2012) was used to check the accuracy of the assembly. The complete chloroplast genome of *O. corniculata* had been submitted to GenBank with the accession number MT648826.

The chloroplast genome of *O. corniculata* has a total length of 152,146 bp with an overall GC content of 36.7%. It exhibits a canonical quadripartite structure including a large single copy (LSC) region of 83,936 bp, a small single copy (SSC) region of 17,048 bp and a pair of 25,581 bp inverted repeats (IRs). The genome encodes 108 unique genes, including 76 protein-coding genes (PCGs), 28 tRNA genes and four rRNA genes.

To reveal the phylogenetic position of *O.corniculata*, we constructed a maximum-likelihood (ML) phylogenetic tree by RAxML v.8.2.10 (Stamatakis 2014), which was based on 11 complete chloroplast genome sequences from the GenBank, including 8 Oxalidales species and 3 Malpighiales species as the outgroup. All the sequences were aligned by MAFFT v.7.308 (Katoh and Standley 2013) with default parameters. As shown in Figure 1, our research illustrated that the chloroplast sequence of *O. corniculata* in this study was clustered closely together (96.2% identity) with the previously reported sequence of *O. corniculata* (Lubna et al. 2020). However, there were 5798 polymorphic sites between two chloroplast sequences of *O. corniculata* (1300 SNPs and 4498 InDels). In addition, *O. corniculata* was grouped with other species of the genus *Oxalis* such as *O. corymbosa* and *O. drummondii*. The complete chloroplast genome sequence of *O. corniculata* will provide an available resource for molecular identification work in this species, and contribute to the phylogenetic analysis for *Oxalis* genus in the future.

**Figure 1.**
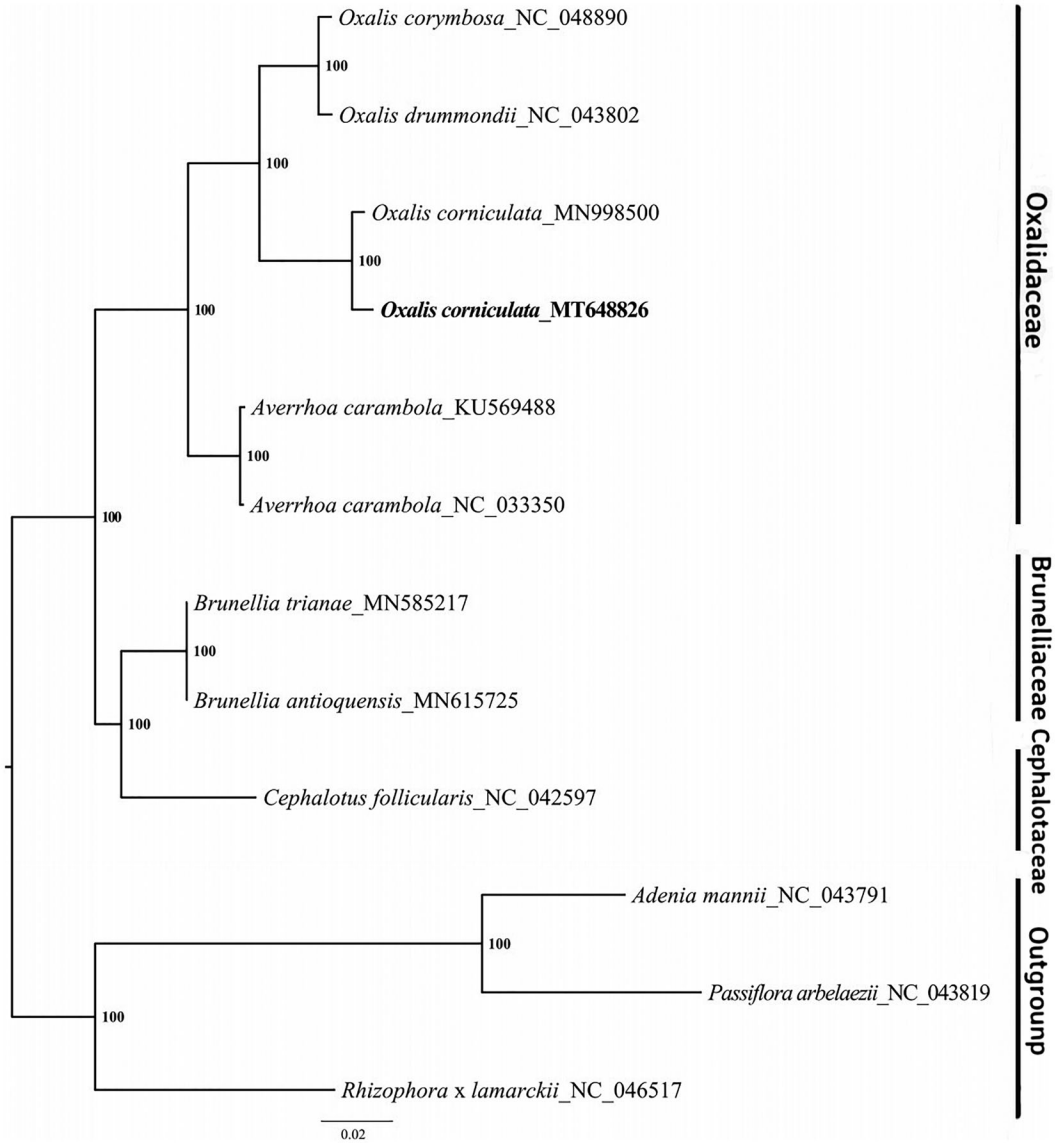
Maximum-likelihood (ML) phylogenetic tree based on complete chloroplast genome sequences of *O. corniculata*, five other Oxalidaceae species, two Brunelliaceae species, one Crephalotaceae species and three Malpighiales species as the outgroup. Numbers on the nodes are bootstrap values from 1000 replicates.

## Data Availability

The data that support the findings of this study are openly available in GenBank of National Center for Biotechnology Information (NCBI) at https://www.ncbi.nlm.nih.gov/, reference number MT648826. The associated BioProject, SRA, and Bio-Sample numbers are PRJNA659844, SRR13123621, and SAMN15929318 respectively.
